# Nationaler Delphi‐Konsens zu Laser‐ und Energie‐basierten Behandlungen der Rosazea

**DOI:** 10.1111/ddg.15976_g

**Published:** 2026-08-04

**Authors:** Lynhda Nguyen, Nikolaus Seeber, Uwe Paasch, Martin Schaller, Claudia Borelli, Syrus Karsai, Gerd Kautz, Tanja C. Fischer, Michael Drosner, Stefan Hammes, Konstantin Feise, Christian Raulin, Martina Kerscher, Gerd Gauglitz, Jens M. Baron, Stefan W. Schneider, Katharina Herberger

**Affiliations:** ^1^ Abteilung für Laser und Ästhetik Klinik und Poliklinik für Dermatologie und Venerologie Universitätsklinikum Hamburg‐Eppendorf, Hamburg; ^2^ Hautarztpraxis Dres. Peter/Seeber/Altheide, Hamburg; ^3^ Klinik und Poliklinik für Dermatologie Venerologie und Allergologie Universitätsklinikum Leipzig; ^4^ Hautklinik Universitätsklinikum Tübingen; ^5^ Abteilung für Laser und Ästhetik Hautklinik Eberhard‐Karls‐Universität Tübingen; ^6^ Dermatologikum Hamburg; ^7^ Haut‐ und Laserklinik Dr. Kautz, Konz; ^8^ Haut‐ und Lasercentrum, Potsdam; ^9^ Haut & Laser Schwerin, Schwerin; ^10^ Klinik und Poliklinik für Mund‐ Kiefer‐ und Gesichtschirurgie Universitätsmedizin Greifswald; ^11^ Sophienklinik, Stuttgart; ^12^ Laserklinik Karlsruhe GmbH, Karlsruhe; ^13^ Abteilung Kosmetikwissenschaften Fachbereich Chemie Universität Hamburg; ^14^ Haut‐ und Laserzentrum Glockenbach, München; ^15^ Klinik für Dermatologie und Allergologie Uniklinik RWTH Aachen; ^16^ Klinik und Poliklinik für Dermatologie und Venerologie Universitätsklinikum Hamburg‐Eppendorf, Hamburg

**Keywords:** Delphi‐Konsens, Energie‐basierte Systeme, Laser, nicht‐ionisierende Strahlquellen, Rosazea, Delphi consensus, energy‐based systems, lasers, non‐ionizing beam sources, rosacea

## Abstract

**Hintergrund:**

Für die Rosazea‐Behandlung steht ein breites Spektrum an Therapieoptionen zur Verfügung, darunter auch Laser‐ und Energie‐basierte Systeme (energy‐based devices, EBDs). Die konkrete Rolle dieser Verfahren für die Rosazea ist in Deutschland bislang jedoch nur unzureichend definiert. Das Ziel dieser Arbeit war die Erarbeitung eines fundierten Expertenkonsenses zum Einsatz von Lasern und EBDs in der Rosazea‐Therapie.

**Patienten und Methodik:**

Die Konsensusfindung erfolgte in drei Phasen. Zunächst wurde eine systematische Literaturübersicht mit Metaanalyse durchgeführt. Anschließend wurden in einer prospektiven Online‐Befragung patientenberichtete Erfahrungen und Sichtweisen zur Behandlung erhoben. In einem dritten Schritt wurde ein strukturierter Delphi‐Prozess über drei Runden durchgeführt, um auf Basis der Literatur und der Patientenangaben Konsensaussagen zu formulieren.

**Ergebnisse:**

An der Befragung nahmen 185 Patienten teil. Unter Einbeziehung der systematischen Übersichtsarbeit und der Patientenrückmeldungen konnte Konsens erzielt werden zu *(1)* der Rolle von Lasern und EBDs bei unterschiedlichen Rosazea‐Phänotypen, *(2)* der Einbindung dieser Verfahren in Kombinationstherapien sowie *(3)* Empfehlungen für eine prä‐ und posttherapeutische Versorgung. Insgesamt beteiligten sich 16 Dermatologen am Delphi‐Prozess.

**Schlussfolgerungen:**

Der vorliegende Expertenkonsens liefert praxisorientierte Empfehlungen für den Einsatz von Laser‐ und EBD‐Therapien in der Rosazea‐Behandlung. Der Delphi‐Prozess verdeutlicht zentrale Aspekte ihres Stellenwerts im Management, ihre Integration in Kombinationstherapien sowie Perspektiven für zukünftige Forschungsansätze.

## EINLEITUNG

Die Rosazea ist eine weit verbreitete, chronisch‐entzündliche Hauterkrankung, die vorwiegend die zentrofaziale Region einschließlich Wangen, Nase, Stirn und Kinn betrifft. Sie zeichnet sich durch ein variables klinisches Erscheinungsbild aus, das häufig mit vorübergehendem Flush beginnt und in ein persistierendes Erythem sowie Teleangiektasien übergeht. Im Verlauf können zusätzlich entzündliche Läsionen wie Papeln und Pusteln auftreten; in manchen Fällen kommt es zu phymatösen Veränderungen.^1^ Neben den sichtbaren Hautmanifestationen berichten Betroffene häufig über sensorische Symptome wie Brennen, Stechen, Trockenheit und Juckreiz, die die Krankheitslast zusätzlich erhöhen. Über die physischen Symptome hinaus wirkt sich Rosazea auch erheblich auf das psychische Wohlbefinden und die Lebensqualität der Patienten aus. Studien zeigten, dass die chronische Erkrankung häufig mit emotionaler Belastung, sozialer Angst und depressiven Symptomen einhergeht.^2^, ^3^

Für die Behandlung der Rosazea steht ein breites Spektrum an Therapieoptionen zur Verfügung, darunter topische und systemische Pharmakotherapien, Laser‐ und Energie‐basierte Systeme (engl.: Energy‐based devices, EBDs), individuell abgestimmte dermokosmetische Behandlungen sowie Anpassung des Lebensstils. Die aktuelle deutsche Leitlinie zur Rosazea (2022) bietet hierzu einen umfassenden Überblick.^4^ Dennoch ist die Rolle von Lasern und EBDs im therapeutischen Algorithmus bislang nur unzureichend definiert, obwohl die Evidenz für ihren Nutzen stetig wächst.^5^, ^6^, ^7^, ^8^ Dennoch ist die Rolle von Lasern und EBDs im therapeutischen Algorithmus bislang nur unzureichend definiert, obwohl die Evidenz für ihren Nutzen stetig wächst. Während pharmakologische Behandlungen entzündliche Läsionen häufig wirksam kontrollieren, reichen sie nicht aus, um ein persistierendes Erythem und Teleangiektasien zu behandeln – Symptome, die von Betroffenen oft als besonders belastend empfunden werden.^9^

Laser‐ und EBD‐Verfahren bieten einen gezielten, nichtinvasiven Ansatz zur Behandlung vaskulärer und entzündlicher Komponenten der Rosazea. Verschiedene Modalitäten, darunter Farbstofflaser (FSL), intensiv gepulstes Licht (IPL) und Neodym‐dotierter Yttrium‐Aluminium‐Granat (Nd:YAG)‐Laser, haben ihre Wirksamkeit bei der Reduktion von Erythem, vaskulären Läsionen und der allgemeinen Krankheitsaktivität gezeigt.^10^ Einheitliche Standards zur Anwendung fehlen jedoch bislang – bedingt durch Unterschiede in der Krankheitsausprägung, in Behandlungsprotokollen, Geräteeinstellungen und Kriterien der Patientenauswahl. Dieses Fehlen klarer Konsenslinien führt sowohl bei Behandelnden als auch bei Patientinnen und Patienten zu Unsicherheiten hinsichtlich des Stellenwerts dieser Technologien.

Angesichts der Diskrepanz zwischen der zunehmenden klinischen Evidenz und den bisherigen Leitlinienempfehlungen besteht ein dringender Bedarf an einem standardisierten, evidenzbasierten Vorgehen zur Integration von Laser‐ und EBD‐Therapien in das Rosazea‐Management. Die vorliegende Konsensusarbeit basiert auf einem dreistufigen Entwicklungsprozess, bestehend aus einer Metaanalyse, einer nationalen Patientenbefragung sowie einem mehrstufigen Delphi‐Expertenkonsens. Ziel war es, ein umfassendes Konsenspapier zur Rolle von Lasern und EBDs in der Rosazea‐Therapie zu erarbeiten. Durch die Schließung bestehender Wissenslücken und die Definition von Best‐Practice‐Empfehlungen soll diese Arbeit dazu beitragen, Behandlungsstrategien zu optimieren, Patientenergebnisse zu verbessern und Ärzten eine praxisnahe Orientierung für den Einsatz dieser modernen Therapieoptionen zu bieten.

## MATERIAL UND METHODIK

### Studiendesign

Die Studie erfolgte in Übereinstimmung mit den Vorgaben des *Acurate Consensus Reporting Document* (ACCORD). Weitere Details sind im Online‐Supplement S1 aufgeführt. Das Forschungsvorhaben war als dreiphasige Studie konzipiert: Zunächst führte eine Koordinierungsgruppe eine umfassende Literaturübersicht mit Metaanalyse durch. Weiterhin wurde eine prospektive, multizentrische Befragung mithilfe eines Online‐Fragebogens durchgeführt, um Erfahrungen und Perspektiven von Rosazea‐Patienten zu erfassen. Schließlich fand ein strukturierter, elektronisch gestützter Delphi‐Prozess über drei Runden statt, mit dem die aus Literatur und Patientenrückmeldungen abgeleiteten Konsensaussagen präzisiert und validiert wurden. Die Umsetzung erfolgte über eine institutionsbasierte Softwareplattform (LimeSurvey V6.1.8, Hamburg, Deutschland) im Zeitraum von Mai 2023 bis August 2024.

### Koordinierungsgruppe und Literaturrecherche

Vor der Formulierung der Konsensaussagen wurde eine systematische Literaturrecherche in *Medline*, *Web of Science* und im *Cochrane Central Register of Controlled Trials* (CENTRAL) durchgeführt. Dabei wurden folgende Suchbegriffe verwendet:

#1 rosacea AND laser*
#2 rosacea AND photodynamic therapy
#3 rosacea AND flash lamp
#4 rosacea AND intense pulsed light
#5 rosacea AND radiofrequency

Zusätzlich erfolgten Vorwärts‐ und Rückwärtssuche, um weitere relevante Studien zu identifizieren. Laufende klinische Studien wurden in der *World Health Organization Trial Registry* sowie auf *ClinicalTrials.gov* recherchiert. Die identifizierten Publikationen wurden systematisch ausgewertet und flossen in eine publizierte Netzwerk‐Metaanalyse ein, die Wirksamkeit und Sicherheit verschiedener laser‐ und lichtbasierter Therapien bei der Rosazea untersuchte.^11^

### Patientenfragebogen

Um Patientenperspektiven neben wissenschaftlicher Expertise einzubeziehen, wurden alle teilnehmenden Experten gebeten, ihre Rosazea‐Patienten zur anonymen Teilnahme an einer Online‐Befragung einzuladen. Der Fragebogen ist im Online‐Supplement S2 enthalten. Darüber hinaus wurde die nationale Patientenorganisation *Deutsche Rosazea Hilfe e.V*. eingeladen, ihre Mitglieder in die Umfrage einzubeziehen.

### Auswahl des Panels

In Deutschland tätige Fachärzte für Dermatologie mit mindestens einer Erst‐ oder Seniorautorschaft im Themenbereich oder mit Beteiligung an relevanten medizinischen Leitlinien wurden zur Teilnahme am Konsensuspanel eingeladen. Um eine größere Diversität und eine breitere Zusammensetzung des Panels zu gewährleisten, wurden anschließend weitere Experten hinzugezogen. Mitglieder der Koordinierungsgruppe fungierten nicht als Panelteilnehmende.

### Modifizierter Delphi‐Konsens

Es wurde eine modifizierte Delphi‐Konsensus‐Methodik angewendet. Auf Grundlage der Literaturrecherche und des Patientenfragebogens identifizierte die Koordinierungsgruppe zentrale Themenbereiche, für die ein nationaler Konsens erforderlich war: *(1)* die Rolle von Lasern und EBD bei der Behandlung unterschiedlicher Rosazea‐Phänotypen, *(2)* der Einsatz von Kombinationstherapien unter Einbeziehung von Laser‐ und EBD‐Behandlungen sowie *(3)* Best‐Practice‐Empfehlungen für die prä‐ und posttherapeutische Versorgung bei Laser‐ und EBD‐Verfahren. Die Koordinierungsgruppe formulierte zunächst eine Reihe vorselektierter Aussagen mit Multiple‐Choice‐Antwortoptionen. Den Panelmitgliedern wurde die Möglichkeit gegeben, über ein optionales Freitextfeld zusätzliche Kommentare oder Änderungsvorschläge einzubringen. Sämtliche Aussagen wurden dem Expertengremium per E‐Mail übermittelt. Integrierte Erinnerungsbenachrichtigungen sollten die Abbruchrate minimieren. Die Panelmitglieder wurden gebeten, zu jeder Aussage anzugeben, ob sie zustimmen, nicht zustimmen oder sich der Stimme enthalten. Während des gesamten Prozesses wurde Anonymität gewährleistet, sodass die Panelmitglieder weder die Identität noch die individuellen Antworten der anderen Teilnehmenden kannten.

### Statistische Analyse

Die statistische Auswertung erfolgte mit SPSS Statistics (IBM, Version 29.0.2.0). Die Darstellung der Teilnehmercharakteristika erfolgte deskriptiv. Es wurden Mittelwerte, Standardabweichungen sowie Spannweiten (Minimum–Maximum) angegeben. Ein p‐Wert von < 0,05 wurde als statistisch signifikant gewertet. Die Zustimmungsraten zu den einzelnen Delphi‐Aussagen wurden in Prozent angegeben. Aussagen mit einer Zustimmung bzw. Ablehnung von ≥ 75 % galten als konsentiert und wurden nicht in nachfolgenden Runden berücksichtigt. Aussagen, die kein Konsens erreichten, wurden auf Grundlage der Rückmeldungen überarbeitet und in der nächsten Runde erneut bewertet.

## ERGEBNISSE

### Patientenbefragung

#### Grundlegende Charakteristika

Insgesamt nahmen 185 Patienten an dem onlinebasierten Fragebogen teil. Das Durchschnittsalter der Teilnehmenden betrug 53,5 ± 11,7 Jahre (27–81), die Mehrheit war weiblich (78,7 %). Im Mittel erhielten die Patienten 5,1 ± 5,5 Lasersitzungen (1–40). Von den Teilnehmern berichteten 42,5 %, ihre Lasertherapie abgeschlossen zu haben, mit durchschnittlich 3,3 ± 2,8 Sitzungen (1–12).

#### Psychosoziale Auswirkungen

Die psychosoziale Belastung durch die Rosazea wurde anhand einer sechsstufigen Skala bewertet (1 = keine, 6 = schwer). Der negative Einfluss der Erkrankung auf das tägliche Leben lag im Mittel bei 3,9 ± 1,5 (1–6), das Erleben von Stigmatisierung bei 2,4 ± 1,5 (1–6). Viele Patienten (64,4 %) berichteten über Einschränkungen im Alltag aufgrund der Rosazea. Am häufigsten genannt wurden Einschränkungen durch den Verzicht auf Saunabesuche (65,0 %), sportliche Aktivitäten (78,6 %), soziale Interaktionen mit Alkoholkonsum (22,3 %) sowie den Verzehr scharfer Speisen (14,6 %).

Bezüglich der primären Ziele einer Lasertherapie gaben die meisten Patienten (82,0 %) die Reduktion von Teleangiektasien an, gefolgt von einer Besserung des persistierenden Erythems (70,1 %). Weitere Ziele waren die Behandlung transienter Erytheme (50,1 %), Rosazea‐assoziierter Ödeme (20,8 %) und Brennen (20,2 %).

#### Verträglichkeit, Sicherheit und Zufriedenheit mit der Behandlung

Die Schwere von Nebenwirkungen wie Schwellung, Rötung und Krustenbildung wurde auf einer sechsstufigen Skala mit durchschnittlich 3,1 ± 1,4 (1–6) bewertet. Die Ausfallzeit nach der Laserbehandlung variierte deutlich zwischen den Patienten und lag im Mittel bei 1,6 ± 3,3 Tagen (0–22,5). Trotz dieser Nebenwirkungen wurde die Lasertherapie insgesamt gut vertragen; die Verträglichkeit wurde mit 4,5 ± 1,4 (1–6) bewertet. Auch die Zufriedenheit mit den Behandlungsergebnissen war hoch, mit einem Durchschnittswert von 4,1 ± 1,8 (1–6). Eine Zusammenfassung der Fragebogenergebnisse findet sich im Online‐Supplement S3.

### Delphi‐Konsens

#### Panelmitglieder

Der Bedarf für eine nationale Konsensstudie zu Laser‐ und energiebasierten Verfahren in der Rosazea‐Therapie wurde erstmals 2022 auf der Jahrestagung der *Deutschen Dermatologischen Lasergesellschaft* (DDL) erkannt. Führende Mitglieder der Fachgesellschaft sowie Experten im Rosazea‐Management wurden beauftragt, evidenzbasierte Empfehlungen für Laser‐ und energiebasierte Verfahren in der Rosazea‐Behandlung zu erarbeiten. Von 23 eingeladenen Fachärztinnen und Fachärzten für Dermatologie erklärten sich 16 zur Teilnahme bereit (bei 7 keine Rückmeldung). Im Verlauf von 16 Monaten interaktiven wissenschaftlichen Austauschs und Abstimmungen wurde ein Konsens erzielt.

#### Delphi‐Runden und Konsensusprozess

**ABBILDUNG 1:**
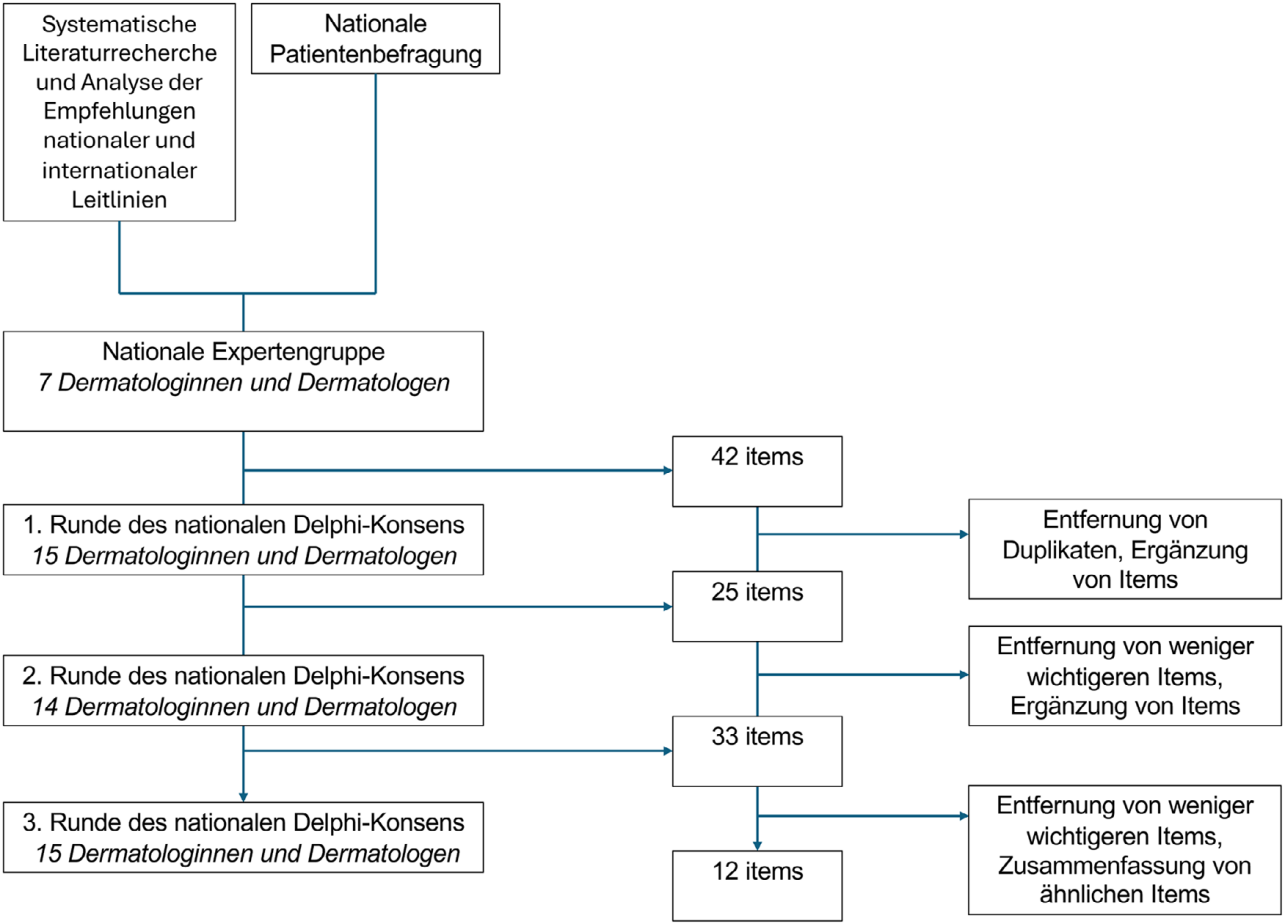
Arbeitsablauf zur Entwicklung der Konsensus‐Statements zum Einsatz von laser‐ und energiebasierten Geräten in der Behandlung der Rosazea.

Der Konsensusprozess wurde in drei aufeinanderfolgenden Runden durchgeführt. Die erste Runde umfasste 25 Aussagen, die zweite 33 und die dritte zwölf Aussagen. Die erste Runde begann im Mai 2023, die zweite folgte im August 2023, und die abschließende dritte Runde wurde im August 2024 abgeschlossen (Abbildung 1).

Die Delphi‐Aussagen wurden in drei Themenbereiche gegliedert: Tabelle 1 enthält Empfehlungen zu Laser‐ und ‐Licht‐basierten Therapien für unterschiedliche Rosazea‐Phänotypen, Tabelle 2 gibt Empfehlungen zu Kombinationstherapien, und Tabelle 3 fasst Empfehlungen zur prä‐ und posttherapeutischen Versorgung im Zusammenhang mit Laser‐ und Energie‐basierten Verfahren bei Rosazea zusammen.

**TABELLE 1:** Konsensus‐Statements zu Laser‐ und Energie‐basierten Behandlungen bei unterschiedlichen Rosazea‐Phänotypen.

Nr.	Statements	Stärke	Zustimmung
1	Zur symptomatischen Behandlung des transienten, zentrofazialen Erythems der Rosazea können Laser oder EBDs empfohlen werden. Dabei sollte das Erythem bei der Behandlung provozierbar sein.	**↑**	84,6 %
2	Zur symptomatischen Behandlung des persistierenden, zentrofazialen Erythems der Rosazea sollen Laser oder EBDs eingesetzt werden.	**↑↑**	92,3 %
3	Zur symptomatischen Behandlung der Teleangiektasien der Rosazea sollen Laser oder EBDs empfohlen werden.	**↑↑**	100 %
4	Zur symptomatischen Behandlung der entzündlichen Komponente der Rosazea können Laser oder EBDs als ergänzende Therapieoption empfohlen werden.	**↑**	92,3 %

**TABELLE 2:** Konsensus‐Statements zu Kombinationstherapien mit Laser‐ und Energie‐basierten Behandlungen bei Rosazea‐Phänotypen.

Nr.	Statements	Stärke	Zustimmung
1	Bei schweren oder therapieresistenten Formen des transienten Erythems der Rosazea kann eine Kombination von topischem Brimonidin, topischem Metronidazol oder topischem Ivermectin mit Lasern oder EBDs empfohlen werden.	**↑**	100 %
2	Bei schweren oder therapieresistenten Formen des persistierenden Erythems der Rosazea kann zusätzlich zu Laser oder EBDs eine Therapie mit topischem Brimonidin, topischem Metronidazol oder topischem Ivermectin empfohlen werden.	**↑↑**	100 %
3	Bei schweren oder therapieresistenten Formen der entzündlichen Komponente der Rosazea kann zur Kombinationstherapie aus einem topischen Wirkstoff (Ivermectin, Metronidazol, Azelainsäure) und einem systemischen Wirkstoff (Doxycyclin, Isotretinoin) zusätzlich eine Therapie mit Laser oder EBDs empfohlen werden.	**↑**	76,9 %
4	Zur Behandlung von Phymata soll eine Therapie mit Laser oder EBDs empfohlen werden. Zusätzlich kann eine Therapie mit systemischem Low‐Dose‐Isotretinoin oder Doxycyclin oder mit topischem Metronidazol empfohlen werden.	**↑↑**	84,6 %

**TABELLE 3:** Konsensus‐Statements zur Vor‐ und Nachsorge bei Laser‐ und Energie‐basierten Behandlungen der Rosazea.

Nr.	Statements	Stärke	Zustimmung
1	Es soll empfohlen werden, die zu betreffenden Regionen vor und nach einer Therapie mit Laser oder EBDs vor der UV‐Strahlung zu schützen.	**↑↑**	100 %
2	Vor und nach einer Therapie mit Laser oder EBDs kann eine Pausierung von topischen Retinoiden und topischer Azelainsäure empfohlen werden.	**↑**	76,9 %
3	Systemische Retinoide stellen keine Kontraindikation für Laser‐ und Energie‐basierte Behandlungen der Rosazea dar.	**↑↑**	100 %

## DISKUSSION

Das primäre Ziel dieses modifizierten Delphi‐Konsensus war es, die Rolle von Lasern und EBDs in der Behandlung der Rosazea zu bewerten und zu definieren. Durch ein strukturiertes und systematisches Vorgehen sollte ein evidenzbasiertes und praxisnahes Empfehlungspapier erarbeitet werden, das die Integration dieser Verfahren in das klinische Management ermöglicht. Neben der Bewertung ihrer Wirksamkeit bei unterschiedlichen Rosazea‐Phänotypen befasste sich der Konsens auch mit ihrem Einsatz in Kombinationstherapien sowie mit Aspekten der prä‐ und posttherapeutischen Versorgung.

Diese Arbeit knüpft an die aktuelle deutsche Rosazea‐Leitlinie an, in der Laser‐ und EBD‐Verfahren bislang als „alternative Behandlungsoptionen“ eingestuft werden.^4^ Diese Klassifizierung erkennt zwar das therapeutische Potenzial an, verdeutlicht jedoch gleichzeitig die Notwendigkeit einer klareren Definition hinsichtlich Wirksamkeit, Sicherheit und Stellenwert in der klinischen Praxis. Mit der Schließung dieser Lücke liefert der vorliegende Konsens ein strukturierteres Rahmenkonzept für den Einsatz dieser Verfahren im Rosazea‐Management.

Zur Einbeziehung der Patientenperspektive wurde eine Online‐Umfrage durchgeführt, die sowohl die psychosoziale Belastung durch die Rosazea als auch die Erfahrungen von Patientinnen und Patienten mit Laser‐ und EBD‐Behandlungen in Deutschland erfasste. Die Ergebnisse bestätigten die erhebliche Beeinträchtigung der Lebensqualität, wobei viele Betroffene Stigmatisierung und soziale Einschränkungen angegeben haben. Im Mittel erhielten die Patienten etwa fünf Behandlungssitzungen. Mehr als die Hälfte berichtete, dass ihre Therapie noch nicht abgeschlossen sei. Moderate Nebenwirkungen wie Erythem, Schwellung und Krustenbildung wurden häufig genannt, waren jedoch vorübergehend und bildeten sich in der Regel innerhalb weniger Tage zurück. Bemerkenswert war das hohe Maß an Zufriedenheit mit den Behandlungsergebnissen, was die Wirksamkeit von Laser‐ und EBD‐Verfahren zur Behandlung Rosazea‐assoziierter Beschwerden unterstreicht.

Die vorliegende Literatur und Expertenmeinungen befürworten den Einsatz von Laser‐ und EBD‐Therapien insbesondere bei persistierendem zentrofazialem Erythem und Teleangiektasien.^4^, ^12^ Auch wenn transiente Erytheme bisher seltener untersucht wurden, deutet der Expertenkonsens darauf hin, dass auch diese durch gezielte Laser‐ und EBD‐Interventionen verbessert werden können, insbesondere wenn das Erythem während der Behandlung provoziert werden kann. Grundlage dieser Verfahren ist das Prinzip der selektiven Photothermolyse, das eine präzise Zerstörung dilatierter Blutgefäße bei minimaler Schädigung des umgebenden Gewebes ermöglicht.^13^

Bei entzündlichen Läsionen im Rahmen der Rosazea können Laser‐ und EBD‐Behandlungen zusätzliche Vorteile bieten, wenn sie in Kombination mit topischen oder systemischen Pharmakotherapien angewendet werden. Kombinationsansätze werden insbesondere bei schweren oder therapieresistenten Verläufen empfohlen, wobei physikalische Verfahren wie Laser und EBDs eine wertvolle Ergänzung darstellen.^4^ Allerdings besteht weiterer Forschungsbedarf, um effektive Kombinationen und Behandlungsprotokolle für die verschiedenen Rosazea‐Phänotypen zu definieren.

Ein weiterer wesentlicher Aspekt der Laser‐ und EBD‐Therapie bei der Rosazea ist die adäquate prä‐ und posttherapeutische Versorgung. Hierbei ist insbesondere der UV‐Schutz von zentraler Bedeutung, da eine UV‐Exposition das Risiko postinflammatorischer Hyperpigmentierungen, Hypopigmentierungen und Verbrennungen erhöht. Patienten, die Laser‐ oder EBD‐Behandlungen erhalten, müssen daher umfassend über die Bedeutung eines konsequenten UV‐Schutzes aufgeklärt werden, um optimale Ergebnisse zu erzielen und Nebenwirkungen zu minimieren.

Ein kontrovers diskutiertes Thema ist der Einsatz von Lasern und EBD bei Patienten unter systemischer Isotretinointherapie. Isotretinoin galt lange Zeit als Kontraindikation für Laserbehandlungen, da eine verzögerte Wundheilung sowie ein erhöhtes Risiko für Narben‐ oder Keloidbildung befürchtet wurden. Diese Annahme stützte sich vor allem auf Berichte aus den 1980er‐ und 1990er‐Jahren, in denen zwei Einzelfälle von Keloidbildung nach einem Argon‐ und Farbstofflaser dokumentiert wurden.^14^, ^15^ Neuere Daten legen jedoch nahe, dass Isotretinoin nicht länger als absolute Kontraindikation gilt. Bei Einhaltung geeigneter Sicherheitsprotokolle können Laser‐ und EBD‐Verfahren auch unter Isotretinointherapie sicher durchgeführt werden.^16^ Nach Expertenkonsens wird empfohlen, die Anwendung von topischem Isotretinoin und Azelainsäure vor und nach Laser‐ oder EBD‐Behandlungen vorübergehend zu pausieren, um potenzielle Irritationen und Hautreaktionen zu minimieren.

Diese Studie weist einige Limitationen auf. Die Mehrheit der Konsensusmitglieder waren Laserdermatologen mit spezieller Expertise in diesem Bereich, was zu einem Bias führen könnte. Um ein ausgewogeneres Bild zu gewährleisten, wurden zusätzliche Rosazea‐Experten in das Panel aufgenommen. Dennoch sollten zukünftige Arbeiten eine breitere interdisziplinäre Beteiligung anstreben, um die Empfehlungen noch umfassender abzusichern. Darüber hinaus konzentrierte sich der vorliegende Konsens ausschließlich auf die Laser‐ und Energie‐basierte Verfahren, die aktuell in den Leitlinien enthalten sind. Künftige Forschung sollte diese Diskussion auf neue therapeutische Ansätze wie die kalte physikalische Plasma‐Therapie ausweiten.

Der Konsens dient als praxisorientiertes Instrument für Ärzte im Rosazea‐Management, insbesondere im Hinblick auf die Integration von Laser‐ und EBD‐Therapien in individualisierte Behandlungspläne. Zusammenfassend adressiert diese Delphi‐Studie zentrale Aspekte der Laser‐ und EBD‐Behandlung bei Rosazea, die aufgrund neuer wissenschaftlicher Erkenntnisse und technologischer Entwicklungen einer fachlichen Konsensbildung bedurften. Die Ergebnisse unterstreichen die Bedeutung individualisierter Therapiestrategien und heben den Stellenwert dieser Verfahren bei der Verbesserung von Therapieergebnissen hervor. Mit dem vorliegenden strukturierten, evidenzbasierten Rahmenkonzept werden Ärztinnen und Ärzte bei fundierten Gesprächen mit Patienten über Wirksamkeit, Sicherheit und praktische Aspekte der Laser‐ und EBD‐Therapien im Rosazea‐Management unterstützt.

## DANKSAGUNG

Wir danken der Patientenvertretung „Deutsche Rosazea Hilfe e.V.“ für die Unterstützung bei dieser Studie.

Open access Veröffentlichung ermöglicht und organisiert durch Projekt DEAL.

## FINANZIERUNG

Diese Studie wurde von der Deutschen Dermatologischen Lasergesellschaft (DDL) unterstützt.

## INTERESSENKONFLIKT

L.N. gibt an, Vortragshonorare von Cynosure Lutronic erhalten zu haben. U.P. berichtet über Forschungsunterstützung und Geräteausleihe von GME, Alma Lasers, Fotona, Zimmer, ThermoCeuticals, Landsberg First Class Aesthetic sowie über Lizenzgebühren von Quintessenz Publishing. C.B. erhält Vortragshonorare von L'Oréal, Asclepion Lasertechnologies, Cutera, Pierre Fabre und P&M Cosmetics. K.F. gibt an, Vortragshonorare von Cutera, Cynosure Lutronic, Palomar und Juzo erhalten zu haben. K.H. hat Vortragshonorare von Cynosure Lutronic erhalten. Alle anderen Autoren geben an, keinen Interessenkonflikt zu haben.

## Supporting information



Supplementary information

Supplementary information
