# Supplementary material for: Nationaler Delphi‐Konsens zu Laser‐ und Energie‐basierten Behandlungen der Rosazea
Source: J Dtsch Dermatol Ges. 2026 Aug 4;24(8):1070–6. [Article in German] doi: 10.1111/ddg.15976_g (PMC13435144; doi:10.1111/ddg.15976_g)
Supplement: Supplementary file 1 [file DDG-24-1070-s001.docx]

|  |   Deutsche Dermatologische Lasergesellschaft e.V. |
| --- | --- |

**ausgefüllt am: ____________**

**Rosazea – Nationaler Konsensus zum Stellenwert der dermatologischen Lasertherapie**

**Patientenfragebogen**

1. **ALLGEMEINES**
a) Alter: _____
b) Geschlecht:	[ ] männlich

[ ] weiblich

[ ] divers

c) Wie viele Laserbehandlungen sind für Ihre Rosazea bereits erfolgt? ________
d) Sind weitere Sitzungen geplant oder ist die Laserbehandlunge bereits abgeschlossen?: [ ] weitere geplant

[ ] abgeschlossen

2. **EINFLUSS AUF DIE LEBENSQUALITÄT**
a) Bitte bewerten Sie Ihre Rosazea-Symptome **vor der Laserbehandlung** auf einer Skala von 0 bis 4:

|  | 0  keine | 1  fast keine | 2  leicht | 3  mittelschwer | 4  schwer |
| --- | --- | --- | --- | --- | --- |
| flüchtige Rötung/ Flushing |  |  |  |  |  |
| andauernde Rötung |  |  |  |  |  |
| erweiterte Gefäße |  |  |  |  |  |
| brennendes Missempfinden |  |  |  |  |  |
| stechendes Missempfinden |  |  |  |  |  |
| Ödem/ Schwellung |  |  |  |  |  |
| Trockenheitsgefühl der Haut |  |  |  |  |  |
| Andere: |  |  |  |  |  |

b) Hat die Rosazea negative Auswirkungen auf ihre Lebensqualität?

| **1**  gar nicht | **2** | **3** | **4** | **5** | **6**  sehr |
| --- | --- | --- | --- | --- | --- |

c) Fühlen Sie sich durch die Rosazea im Alltag belastet?

| **1**  gar nicht | **2** | **3** | **4** | **5** | **6**  sehr |
| --- | --- | --- | --- | --- | --- |

d) Haben Sie aufgrund der Rosazea Erfahrungen mit Stigmatisierung gemacht?

| **1**  gar nicht | **2** | **3** | **4** | **5** | **6**  sehr |
| --- | --- | --- | --- | --- | --- |

e) Haben Sie durch die Rosazea Einschränkungen in Tätigkeiten (z.B. Sauna, Sport,…)?: [ ] ja

[ ] nein

f) Wenn ja, welche Tätigkeiten sind es?
3. **LASERTHERAPIE**
a) Wie ausgeprägt waren die Nebenwirkungen nach der Laserbehandlung (Schwellung, Rötung, Blaufärbung, Krusten) und wie lange hielten sie an?

| **1**  gar nicht | **2** | **3** | **4** | **5** | **6**  sehr |
| --- | --- | --- | --- | --- | --- |

**Dauer:** _____________

b) War die Lasertherapie verträglich?

| **1**  gar nicht | **2** | **3** | **4** | **5** | **6**  sehr |
| --- | --- | --- | --- | --- | --- |

c) Wie lange war Ihre Ausfallzeit durch die Laserbehandlung (in Tagen)?: _________________
d) Wie zufrieden waren Sie mit dem Behandlungsergebnis?

| **1**  gar nicht | **2** | **3** | **4** | **5** | **6**  sehr |
| --- | --- | --- | --- | --- | --- |

e) Kreuzen Sie bitte das von Ihnen gewünschte Ziel der Laserbehandlung an (mehrere Auswahlen möglich):

Verbesserung der…

| - flüchtige Rötung | - andauernde Rötung | - erweiterte Gefäße |
| --- | --- | --- |
| - brennendes Missempfinden | - stechendes Missempfinden | - Ödem/ Schwellung |
| - Trockenheitsgefühl der Haut | - Andere: | |

f) Hat die Behandlung Ihre Ziele erfüllt?

| **1**  gar nicht | **2** | **3** | **4** | **5** | **6**  sehr |
| --- | --- | --- | --- | --- | --- |

g) Bitte bewerten Sie Ihre Rosazea-Symptome **nach der Laserbehandlung** auf einer Skala von 0 bis 4:

|  | 0  keine | 1  fast keine | 2  leicht | 3  mittelschwer | 4  schwer |
| --- | --- | --- | --- | --- | --- |
| flüchtige Rötung/ Flushing |  |  |  |  |  |
| andauernde Rötung |  |  |  |  |  |
| erweiterte Gefäße |  |  |  |  |  |
| brennendes Missempfinden |  |  |  |  |  |
| stechendes Missempfinden |  |  |  |  |  |
| Ödem/ Schwellung |  |  |  |  |  |
| Trockenheitsgefühl der Haut |  |  |  |  |  |
| Andere: |  |  |  |  |  |

h) Welchen Wert würden Sie für das erreichte Behandlungsergebnis für eine Laserbehandlunge als angemessen empfinden?

| **< 50€** | **50 – 100€** | **100 – 150€** | **150 – 200€** | **>200€** |
| --- | --- | --- | --- | --- |

i) Würden Sie die Behandlung weiterempfehlen?

- ja
- nein	► Kommentar:

j) Andere Beschwerden/ Kommentar:
