# Supplementary material for: Nationaler Delphi‐Konsens zu Laser‐ und Energie‐basierten Behandlungen der Rosazea
Source: J Dtsch Dermatol Ges. 2026 Aug 4;24(8):1070–6. [Article in German] doi: 10.1111/ddg.15976_g (PMC13435144; doi:10.1111/ddg.15976_g)
Supplement: Supplementary file 2 [file DDG-24-1070-s002.docx]

| **Domäne** | **Parameter** | **Wert / Statistik** |
| --- | --- | --- |
| **Basischarakteristika**  **Online-Supplement S3** | Anzahl der Teilnehmenden | 185 |
|  | Alter (Mittelwert ± SD, Minimum – Maximum) | 53.5 ± 11.7 Jahre (27 – 81) |
|  | männlich/ weiblich (%) | 21.3/ 78.7 |
|  | Durchschnittliche Anzahl an Sitzungen (Mittelwert ± SD, Minimum – Maximum) | 5.1 ± 5.5 (1 – 40) |
|  | Abgeschlossene Behandlungen (%) | 42.5 % |
|  | Anzahl der Sitzungen bei abgeschlossener Behandlung (Mittelwert ± SD, Minimum – Maximum) | 3.3 ± 2.8 (1 – 12) |
| **Psychosoziale Belastung** | Negativer Einfluss auf das tägliche Leben  Skala von 1 (kein) – 6 (stark) | 3.9 ± 1.5 (1-6) |
|  | Wahrgenommene Stigmatisierung  Skala von 1 (kein) – 6 (stark) | 2.4 ± 1.5 (1-6) |
|  | Einschränkungen im Alltag (%) | 64.4 |
|  | Am häufigsten betroffene Aktivitäten (%) | Saunagänge: 65.0; Sport: 78.6; Alkoholkonsum: 22.3; scharfes Essen: 14.6 |
| **Primäre Ziele der Laserbehandlung** | Reduktion von Teleangiektasien (%) | 82.0 |
|  | Verbesserung des persistierenden Erythems (%) | 70.1 |
|  | Verbesserung des transienten Erythems (%) | 50.1 |
|  | Reduktion Rosazea-assoziierter Ödeme (%) | 20.8 |
|  | Reduktion von Brennen (%) | 20.2 |
| **Verträglichkeit, Sicherheit, Patientenzufriedenheit** | Schweregrad von Nebenwirkungen  Skala von 1 (kein) – 6 (stark) | 3.1 ± 1.4 (1-6) |
|  | Ausfallzeit nach Behandlung (Mittelwert ± SD, Minimum – Maximum) | 1.6 ± 3.3 Tage (0-22.5) |
|  | Verträglichkeit der Behandlung  Skala von 1 (gar nicht) – 6 (sehr) | 4.5 ± 1.4 (1-6) |
|  | Zufriedenheit mit Behandlungsergebnissen  Skala von 1 (gar nicht) – 6 (sehr) | 4.1 ± 1.8 (1-6) |
